# The Reliability of Common Functional Performance Tests within an Experimental Test Battery for the Lower Extremities

**DOI:** 10.3390/sports9070100

**Published:** 2021-07-12

**Authors:** Giordano Scinicarelli, Marko Trofenik, Ingo Froböse, Christiane Wilke

**Affiliations:** Institute of Movement Therapy and Movement-Oriented Prevention and Rehabilitation Sciences, German Sport University, 50933 Cologne, Germany; markotrofenik92@gmail.com (M.T.); Froboese@dshs-koeln.de (I.F.); Wilke@dshs-koeln.de (C.W.)

**Keywords:** test-retest design, limb symmetry index, between-session differences, healthy athletes, dynamic-balance test, hop test, sprint test, hop performance, speed performance

## Abstract

The main aim of this study was to determine the test–retest reliability of an experimental functional test battery: Y-balance test (YBT), single-leg countermovement jump (SLCMJ), single-leg hop for distance (SLH), side-hop (SH), speedy-jump (SJ), agility-T (AT), and lower extremity functional test (LEFT). Secondary aims were (1) to determine the mean range for the limb symmetry index (LSI) and (2) to detect significant differences in performance between test–retest sessions. Twenty-two healthy adults (14 males, 8 females; age 23.3 ± 3.9) were tested by the same rater during two different sessions (T1–T2), with a seven-day interval, under the same conditions. Reliability analysis showed good-to-excellent reliability (0.89 < ICC < 0.97; 0.80 < α < 0.98) for the test battery. LSI ranged from 95.9 ± 6.7% (SH-T1) to 104.4 ± 12.5% (SLCMJ-T2). Repeated measures ANOVA detected significant performance differences between sessions in the LEFT (*p* = 0.009) and for non-dominant sides in the SLH (*p* = 0.015), SH (*p* = 0.007), and SJ (*p* = 0.011). The high reliability of the test battery suggests a potential use in clinical sports practice. The LSI range of ≥95% was proposed as a benchmark for healthy adults. Learning effect seems to have played a crucial role in the T2 improvements of the non-dominant side for hop performances (SLH, SH, SJ) and speed performance (LEFT).

## 1. Introduction

A test battery consists of an evaluated and standardized protocol combining single and complementary subtests, which do not require sophisticated equipment and can be carried out multiple times [1]. Test batteries are an efficient screening tool to assess functional movement patterns [2] as well as to provide a multidimensional, objective, and quantitative analysis for the evaluation of functional performance [3]. In sports therapy, functional test batteries are widely used to assess sport-specific functional performance [2,4], to establish prevention strategies (such as pre-injury screenings) for anterior cruciate ligament injury (ACL-I) [5,6], and to plan specific training interventions for the lower extremities [7,8,9,10]. Furthermore, they are regularly used in monitoring and decision-making processes of rehabilitation after ACL-I, for return-to-sport clearance, and for movement quality assessments [4,11,12,13,14].

In general, functional inter-limb asymmetries in performance are associated with sport activity limitations and might be an injury risk factor for uninjured athletes [2]. Clinically, the limb symmetry index (LSI) can be calculated easily and rapidly. It can provide valuable baseline data for pre-injury screenings or for rehabilitation progression purposes. In order to allow side-to-side performance comparisons, functional tests can be performed unilaterally (on one leg) and the LSI can be detected [15,16,17,18,19]. The LSI is an indicator that quantifies the symmetry of the limbs in percentage and can be calculated in multiple ways: uninjured/injured, non-dominant/dominant, or less-performant/more-performant limbs [20,21]. As a rule, an LSI ≥ 90% is considered a normal range in functional tests for both injured and uninjured populations [4,14,22].

Generally, a valuable test battery should include sport-specific movement patterns (e.g., balancing, landing, sprinting and cutting), in order to provide sport-specific functional performance outcomes in terms of imbalances/weaknesses related to muscle power, dynamic balance, proprioception, speed and agility [2,23,24]. In particular, an effective test battery should encompass several functional tests structured into different levels, gradually organized from simple (less demanding) to complex (more demanding) tests [1,4,14,25,26]. Thus, the design should be primarily based on the assessment of postural control, dynamic balance, and joint stability [4,14,27,28]. Subsequently, measurements of muscular power/strength, proprioception, and neuromuscular control, among which single-leg hop tasks, should be included [4,14,29,30,31]. Finally, agility, speed, and resistance in a fatigue state should also be assessed [1,4,32,33,34,35]. Several studies investigated the reliability of single functional tests in relation to performance differences and future lower extremity injuries [1,4,11,28,29,30,32,33,35,36]. However, various test–retest reliability designs have been used hitherto and a lack of standardized testing protocols and procedures can be found in the literature [37]. For these reasons, carrying out clinical and methodological comparisons might be quite challenging.

In order to be considered available, believable, and informative, a test battery, along with all the included tests, needs to also be reliable. The tests included in this study are all reliable per se. However, their reliability has not yet been investigated in relation to a sequentially ordered, experimental test battery with evidence-based level design and standardized testing procedures, as was the case in the present study. Test–retest reliability refers to the degree of values similarity between two same repeated measurements under the same conditions on the same individuals [38,39,40,41,42,43,44,45,46,47,48].

Therefore, the main objective of this study was to determine the test–retest reliability of an experimental functional test battery with a seven-day interval. A two-fold secondary objective was to establish whether healthy adults showed a limb symmetry index greater than or equal to 90% and to investigate the presence of significant differences between performances of the two testing sessions (T1 and T2). It was hypothesized that: (1) the test battery should demonstrate at least good reliability coefficients (ICC ≥ 0.75; Cronbach’ α ≥ 0.80), and (2) healthy adults should show at least a normal inter-limb asymmetry range (LSI ≥ 90%) in all tests performed and should not present significant differences (*p* > 0.05) in performance between the two testing sessions (T1 and T2).

## 2. Materials and Methods

### 2.1. Study Design

This study was conducted with a test–retest design, which allowed repeated measures for reliability analysis. A test–retest interval should have adequate time to recover between testing sessions as well as reduce the influence of learning effect and of physical/fitness status changes [34,49]. Two identical testing sessions (T1 and T2) were performed with a seven-day interval time under the same conditions: subjects were tested twice by the same rater (a sports physical therapist with six years of scientific experience in the field); the same indoor laboratory was used for sports therapy purposes; and both sessions were performed in the afternoon. For the sake of consistency, a predefined order of the experimental test battery was applied in both testing sessions, as follows: (1) Y-balance test (YBT); (2) single-leg countermovement jump test (SLCMJ); (3) single-leg hop for distance test (SLH); (4) side hop test (SH); (5) speedy jump test (SJ); (6) agility-T Test (AT); and (7) lower extremity functional test (LEFT). A written informed consent was signed by all subjects and the study was approved by the Ethical Committee of the University (056/2018).

### 2.2. Subjects

Twenty-two healthy adults (8 females and 14 males; age 23.3 ± 3.9) participated in the study. Anthropometrics data are presented in Table 1. Subjects were all volunteers and uninjured collegiate student-athletes from the German Sport University of Cologne. The inclusion criteria for participants were (1) age ranging between 18–30 years and (2) active participation in individual or team sports activity without any restrictions in practices (2–4 × week) or games (1 × week) over the previous twelve months. Additionally, all subjects were not affected by any musculoskeletal disease that could have influenced the results. The exclusion criterion for the study was the presence of a lower extremity major injury (>21 days of absence) over the previous twelve months. All subjects were aware of the potential risks and benefits of the study and complied with the design, protocol, and inclusion criteria; no subject was excluded from the study.

### 2.3. Procedures

This study was conducted at the Institute of Movement Therapy and Movement-Oriented Prevention and Rehabilitation Science, at the German Sport University of Cologne. Both testing sessions took place during the winter-break in January 2020. Before performing the test battery, all subjects executed a standardized warm-up program including ten minutes of stationary bike at low intensity and five minutes of guided, lower-limb joint mobility. The unilateral tests (YBT, SLCMJ, SLH, SH, and SJ) were executed barefoot on a therapeutic sport mat (FUCHSIUS^®^ multi-media GmbH, Munich, Germany), with the hands necessarily placed on the hips during the entire execution. The bilateral sprint tests (AT and LEFT) were performed on an athletic indoor track and, therefore, subjects were asked to use their own running shoes. All subjects had to wear only sports t-shirt and shorts while performing the test battery. The limb dominance was determined by the leg with which the subjects would kick a ball [50]. In order to familiarize them with the tests, standardized instructions and demonstrations were provided before each test. For the sake of consistency, all subjects were given two practice trials (one per limb) in the unilateral tests and one practice trial in the bilateral sprint tests. Afterward, three maximum valid attempts were performed for each limb in the unilateral tests while two maximum valid attempts were performed in the bilateral tests. A recovery time of 30 s was allowed between practice/valid attempts, while a recovery time of two minutes was applied between each test. In the unilateral tests, the limb to be tested first was randomly selected in order to avoid learning/fatigue effects. The test rater decided in real-time whether the tests were carried out correctly or not. For each test, the best score amongst the valid attempts was used for the data analysis. If no valid attempt was recorded, the subject had to be excluded from the analysis; however, all subjects recorded at least one valid attempt and, therefore, all subjects were included in the analysis. Finally, verbal encouragement and transcription of the scores took place consistently during the two testing measurements (T1 and T2).

### 2.4. Test Battery Standardization and Description

**Figure 1:**
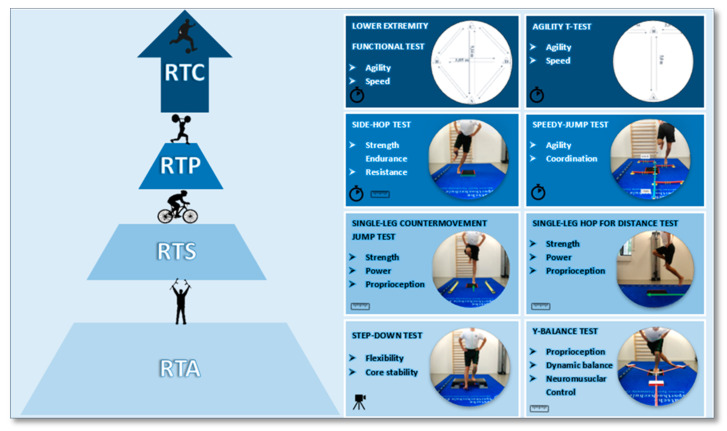
Design of the proposed test battery. Legend for symbols: the camera indicates that only a qualitative analysis of movement is possible (video recording); the meter indicates that a quantitative analysis of the performance can be made (numerical, e.g., in centimeters); the stopwatch indicates that a quantitative analysis of the performance can be made (numerical, e.g., in seconds).

Figure 1 shows the design of the proposed test battery based on the literature. It is divided into four levels: (1) return to activity (RTA); (2) return to sport (RTS); (3) return to play (RTP); and (4) return to competition (RTC). Each level comprises two tests, which are organized from the simplest to the most complex level of difficulty in terms of effort and execution [4,14]. In this context, the following seven tests were selected and included in the present study. The Y-balance test (YBT), a reliable and predictive measure for lower extremity injuries in high school basketball and American football players [28,51], which is also related to chronic ankle instability in normal population [52]. The single-leg countermovement jump test (SLCMJ) is a reliable and appropriate measurement for determining muscle power functions of the lower extremities in healthy or rehabilitated athletes [29] and a useful test for the evaluation of knee function after ACL-reconstruction (ACL-R) [36]. The single-leg hop for distance test (SLH) is a reliable measurement recommended in clinical or sport practice [33]. Additionally, the SLH has proven to be highly valuable in discriminating between injured/uninjured hop performance in patients with an ACL-I/ACL-R [30] and makes it possible to identify competitive athletes at risk for lower back/lower extremity injuries [32]. Furthermore, the SLH is normally used for return-to-sport clearance [4] and has been demonstrated to be a practicable task for the evaluation of knee function after ACL-R [36]. The side-hop test (SH) is a valid and reliable measurement to evaluate knee function after ACL-R [36] and to discriminate between injured/uninjured hop performance in patients with an ACL-I/ACL-R [30]. The speedy-jump test (SJ) is a reliable tool in identifying functional deficits of the knee in clinical environments [1] and to assist the rehabilitation process after ACL-R [11]. The agility T-test (AT) is recommended in clinical and sport practice [33] and is a reliable measurement in determining low or high levels of sports participation in college athletes [35]. The lower extremity functional test (LEFT) is a reliable measurement in identifying competitive athletes at risk for lower back/lower extremity injuries [32]. In the present study, the first level comprised only one test because the step-down test was not performed and not included in the reliability analysis, since it does not provide any quantitative performance data as it is usually used to analyze the quality of movement [53].

#### 2.4.1. Step-Down Test (SD)

According to Park et al., the SD test is performed without shoes and the starting position is on a 20 cm high step. Subjects stand upright on one leg with the toes of the standing leg close to the edge of the step. The free leg is extended in front of the step with the ankle in maximum dorsiflexion. With as much control as possible, subjects are asked to bend the knee of the standing leg until the heel of the extended leg touches the floor and then immediately return to the starting position. During the test execution, the following criteria should be used to mark invalid attempts: single-leg balance is not fully maintained, the trunk is not kept straight, the standing leg does not remain in contact with the step with the whole foot and the hands do not remain fixed at the hips. Subjects have one trial attempt and one valid attempt per leg [53].

#### 2.4.2. Y-Balance Test (YBT)

The YBT is a valid, reliable test to assess postural control and balance capacities [4,14,28]. The Y-Balance Test Kit (Move2Perform^®^, Evansville, IN, USA) was utilized. The subjects started in a standing position on one leg, with the toes of the standing leg positioned at the red line marked on the central platform of the instrument. The sliding elements had to be pushed with the toes of the contralateral leg as far as possible in three directions (anterior, posteromedial, and posterolateral). For correct execution, the standing leg keeps a full stance on the platform and the contralateral leg keeps constant contact with the sliding elements. After that, subjects had to return to the starting point and keep the final balancing position (on one leg) for three seconds (measured with a stopwatch) to be considered as a valid attempt. The following criteria were used to mark invalid attempts: leaving the arms from the hips, loss of balance, contact of the contralateral leg with the ground, lifting up the heel of the standing leg, kicking the sliding element or standing on top of it. For the normalization of the scores, the limb length had to be measured [28]. The performance was then computed as a “composite score” in percentage using this predetermined formula: composite score = (anterior + posteromedial + posterolateral performances in cm) / 3 × limb length in cm) × 100 [28].

#### 2.4.3. Single-Leg Countermovement Jump Test (SLCMJ)

The SLCMJ is a valid and reliable test that measures proprioception and neuromuscular control abilities [4,30]. The OptoJump Kit (Version 1.12.1.0—Microgate^®^, Bolzano, Italy) was utilized. The subjects started in a standing position on one leg, performed a countermovement flexion with the standing leg and then explosively jumped as high as possible [54]. To be considered as a valid attempt, the landing with the same leg had to be maintained stable for three seconds (measured with a stopwatch). The following criteria were used to mark invalid attempts: leaving the arms from the hips, multiple jumps while landing, flexing the jumping leg during the flight phase, a contact with the ground or a swing of the contralateral leg. The vertical jumped height was then measured in centimeters (cm) by using the OptoJump software.

#### 2.4.4. Single Leg Hop for Distance Test (SLH)

The SLH is a valid and reliable test useful to assess muscle strength and power deficits [4,49]. The subjects started in a standing position on one leg, with the toes positioned at the starting line marked on the therapeutic mat. The subjects had to jump as far as possible, landing on the same leg. The landing had to be maintained stable for three seconds (measured with a stopwatch), otherwise, the attempt was marked as invalid. The following criteria were used to mark invalid attempts: leaving the arms from the hips, a swing of the contralateral leg, using the contralateral leg as a support, loss of balance or multiple jumps at landing. The jumped distance was then measured in centimeters (cm) with a measuring tape, from the starting line marked on the mat (jump take-off) to the heel of the subjects where the landing took place [54].

#### 2.4.5. Side-Hop Test (SH)

The SH is a valid and reliable test to assess strength resistance under fatigue state through controlled, fast, and repetitive lateral jumps [29,30]. The subjects started in a standing position on one leg with their hands on the hips, jumping sideways over two parallel lines (40 cm apart) painted on the therapeutic mat. Subjects performed as many jumps as possible in 30 s, recorded using a stopwatch. After the last jump, a controlled landing had to be maintained for three seconds (measured with a stopwatch), otherwise the attempt was marked as invalid. The following criteria were used to mark invalid attempts: jumping on the painted line whit the tested leg, performing extra/double jumps, supporting of the contra-lateral leg, or leaving the arms from the hips [4,29,30]. The number of successful jumps (score = total jumps − error jumps) were counted live by the test leader.

#### 2.4.6. Speedy-Jump Test (SJ)

The SJ is a valid and reliable test to estimate power, dynamic knee stability, and coordination of the lower extremities while jumping as fast as possible through different plane directions [1]. A predetermined Speedy Basic Jump Set (TST—Trendsport^®^, Grosshöflein, Austria) was utilized. Subjects started in a standing position on one leg. The subjects executed three jumps on each of the four red bars (jumping forward, backward, and forward) and one jump on each of the four blue bars (jumping sideway), performing sixteen jumps in total [1]. After the last jump, a controlled landing with the same leg had to be maintained for three seconds (measured with a stopwatch), otherwise the attempt was marked as invalid. The following criteria were used to mark invalid attempts: leaving the arms from the hips, a contact with the test instrument and a swing or ground support with the contralateral leg. The execution time was computed in seconds (s) with a stopwatch, from the moment of the first jump (take-off phase) to the moment of the last jump (landing). 

#### 2.4.7. Agility *T*-Test (AT)

**Figure 2:**
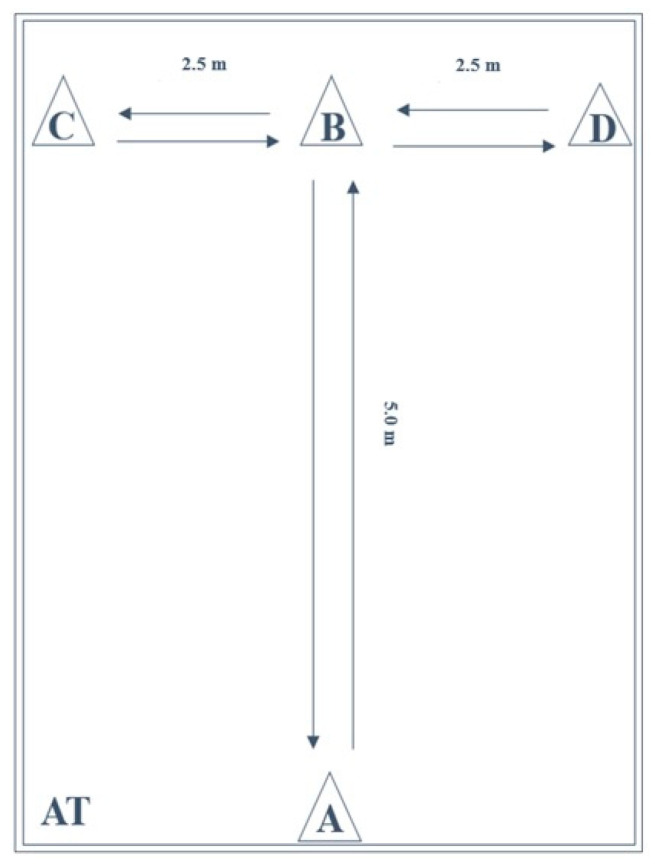
Schematic representation of the agility *T*-test (AT).

The AT (Figure 2) is a valid and reliable test for the measurement of agility and change of direction speed by maximum start, side steps, and running backwards [4,33,35,55,56]. The layout is a combination of four cones in T-shape (5 m × 5 m). Subjects started in a standing position behind the starting point at cone A. After the start signal, subjects sprinted to cone B, touching it with their right hand. Then, they performed a side-shuffle to the left to cone C, touching it with their left hand. Next, they performed a side-shuffle to the right to cone D, touching it with their right hand. Then, they performed a side-shuffle to cone B, touching it with their left hand. After that, they performed a backward run to cone A. Attempts were considered invalid if the subjects did not touch the cones, performed the side-shuffle crossing their legs or did not face forward while sprinting or side-shuffling [4,33,35,55,56]. The execution time was computed in seconds (s) with a stopwatch, from the moment of the first sprint as soon as subjects left cone A to the moment of the last sprint as soon as subjects passed cone A.

#### 2.4.8. Lower Extremity Functional Test (LEFT)

**Figure 3:**
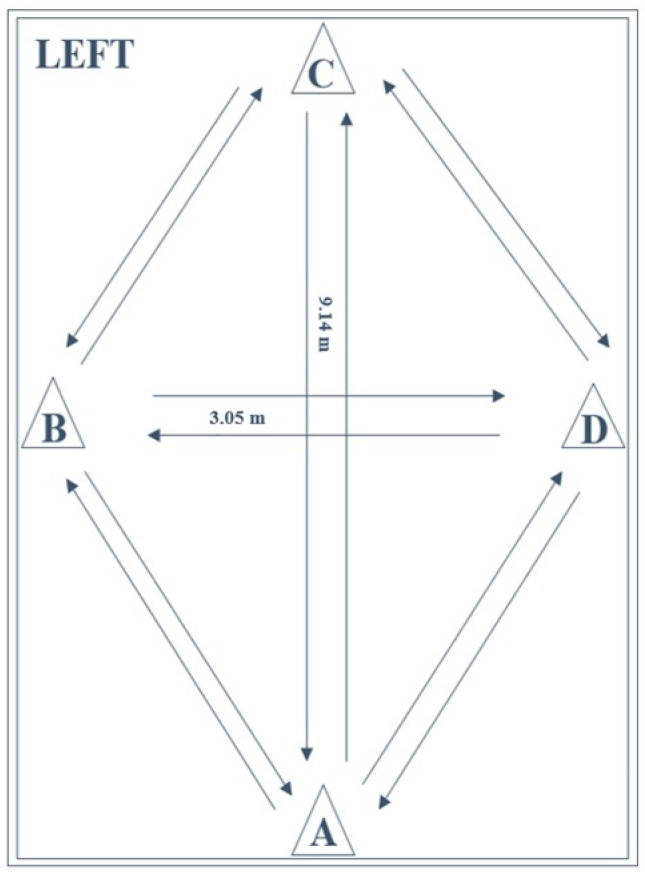
Schematic representation of the lower extremity functional test (LEFT).

The LEFT (Figure 3) is a reliable and valid test for the measurement of athletic fitness, fatigue resistance, and speed by performing a series of 16 specific maneuvers as fast as possible (including forward and backward sprinting, sidestepping, cross-stepping, 45° and 90° cutting) [4,32,34,55]. The layout is a combination of four cones in a diamond-shape (9.14 m × 3.05 m). Test execution was performed in accordance with previously described methods [4,32]. Subjects started in an upright standing position with both feet behind the starting point at cone A. On the command of the instructor, the subjects performed eight different agility tasks, with each task being performed twice (once to the right and once to the left direction). Because of the multidirectional requirements of the test and variety of tasks to be performed, verbal instruction of subsequent movements was provided throughout the test. As such, subjects were required to respond to the external stimuli. Attempts were considered invalid if participants failed to perform the designated maneuvers or dropped a cone by contact. The execution time was computed in seconds (s) with a stopwatch, from the moment of the first sprint after the starting signal as soon as the subjects left cone A to the moment of the last sprint as soon as the subjects passed cone A.

### 2.5. Test–Retest Reliability

The main measures of reliability are the intraclass correlation coefficient (ICC) and Cronbach’s alpha coefficient (α), which were both considered in the present study. The higher the correlation coefficients, the greater the reliability of measurements [38]. The ICC and Cronbach’s α are coefficients ranging from 0 to 1: in general, good coefficients magnitudes (ICC ≥ 0.75; Cronbach’s α ≥ 0.80) are required for a measurement to be considered reliable, while excellent coefficients magnitudes (ICC ≥ 0.90; Cronbach’s α ≥ 0.90) indicate a highly reliable measurement [26,39,40,41,42]. Other adaptable parameters could affect the reliability results and were also considered in the present study, such as sample size heterogeneity, within-subject variations, systematic changes in mean and measurement errors [42,43]. Additionally, attention should be paid to the span of time between the two test measurements as much as to the motor learning effect of the subjects [44,45,46,47]; therefore, both aspects were considered in the present study in order to reduce their influence on the test–retest results. Conversely, gender and sport type seem to have no influence on reliability results, which is why these were not considered in the present study [44,45,46,47]. Various interval times between the test–retest measurements have been used hitherto in the literature, ranging from ten-minute to one-month intervals. Nevertheless, the most used intervals ranged from two days to two weeks [38,48]. In the present study, a seven-day interval was chosen in order to ensure that participants had sufficient time for recovery between sessions and, at the same time, not too long to be able to produce changes in performance related to training. In addition, between the two testing sessions, participants were explicitly asked to avoid practicing the test battery and were allowed to solely perform their usual sports training.

### 2.6. Statistical Analysis

SPSS for Windows Version 26.0 (SPSS Inc., Chicago, IL, USA) was used for all statistical analyses, significance was set at *p* < 0.05 while the limits of agreement were set at a 95% confidence interval (CI 95%). Normality of data was evaluated by the Shapiro–Wilk Test while homogeneity of variance was established with the Levene’s test. Descriptive statistics of anthropometrics were calculated by means and standard deviations (±SD). For each test, mean values and standard deviations (±SD) were calculated from the valid attempt performed with the best score. For the unilateral tests (YBT, SLCMJ, SLH, SH, and SJ), mean values, and standard deviations (±SD) were calculated on three variables: dominant limb, non-dominant limb, and limb symmetry index (LSI). The LSI was calculated using the proposed formula [LSI = (non-dominant/dominant) * 100] for the uninjured population [15,57]. For the bilateral sprint tests (AT and LEFT), mean values, and standard deviations (±SD) were calculated using performance scores on one variable. The tests were analyzed in relation to the following dependent variables: Y-balance test (YBT), dominant/non-dominant composite score (%), and LSI (%); single-leg countermovement jump test (SLCMJ), dominant/non-dominant vertical jumped height (centimeter) and LSI (%); single-leg hop for distance test (SLH), dominant/non-dominant horizontal jumped length (centimeter) and LSI (%); side-hop test (SH), dominant/non-dominant number of jumps (number of jumps) and LSI (%); speedy-jump test (SJ), dominant/non-dominant time of execution (seconds) and LSI (%); agility *T*-test (AT), time of execution (seconds); lower extremity functional test (LEFT), time of execution (seconds). To determine the test–retest reliability of the dependent variables, the intraclass correlation coefficient (ICC) for the reproducibility of quantitative measurements and Cronbach’s alpha (α) for the internal consistency were used. The ICC for the single measures was solely considered since the ratings were performed by a single rater. However, other parameters affect the reliability analysis and were therefore included in this study: the coefficient of variation (CV) for the extent of variability, defined as the ratio of the SD to the mean (CV = SD/Mean × 100); the standard error of measurement (SEM) for the effect of measurement error, defined as the SD of an individual’s repeated measurements (SEM = SD × √1 − ICC); the smallest real difference (SRD), defined as a measure of sensitivity to change (SRD = 1.96 × √2 × SEM) [42,43]. To assess the magnitude of the reliability analysis, the threshold values were considered as follows: poor (<0.5), moderate (0.50–0.75), good (0.75–0.90), and excellent (>0.90) for the ICC [44]; unacceptable (<0.5), poor (0.5–0.6), questionable (0.6–0.7), acceptable (0.7–0.8), good (0.8–0.9), and excellent (>0.90) for the Cronbach’s α [45]; not acceptable (>30), acceptable (20–30), good (10–20) and very good (<10) for the CV [46]; perfectly reliable (equal to 0) and completely unreliable (equal to the SD) for the SEM [46]; acceptable (<30%) for the SRD [47]. Finally, for the analysis of variance of the dependent variables, repeated measures ANOVA (*p* < 0.05) was used separately to compare differences in mean scores between the two testing sessions (T1 and T2). Repeated measures ANOVA was chosen to compare three quantitative dependent variables (dominant, non-dominant and LSI) on the same samples divided per age groups (from U11 to U19), in each of the test performed (except for LEFT and AT, where only one variable was analyzed). It was assumed that the means would have been identical between the two test sessions (T1 and T2). To this end, the within-subjects effect was considered and preferred over the between-subjects effect, since the variances to be analyzed definitely concerned the same subjects, the same leg and the same test, but on two different test occasions across time (T1 and T2).

## 3. Results

The results of the test battery are shown in Table 2. The Shapiro–Wilk test revealed that all data were normally distributed (*p* > 0.05) and the Levene’s test revealed the homogeneity of variance (*p* > 0.05).

As far as reliability analysis is concerned, the intraclass correlation coefficients (ICC) of dominant limbs ranged from good 0.89 (YBT) to excellent 0.97 (SJ) while Cronbach’s α coefficients maintained an excellent range from 0.92 (YBT) to 0.98 (SJ); the intraclass correlation coefficients (ICC) of non-dominant limbs ranged from moderate 0.71 (YBT) to excellent 0.96 (SJ) while the Cronbach’s α coefficients ranged from good 0.80 (YBT) to excellent 0.98 (SJ); the intraclass correlation coefficients (ICC) of the LSI ranged from poor 0.41 (SH) to good 0.76 (SLCMJ) while the Cronbach’s α coefficients ranged from poor 0.50 (SH) to good 0.83 (SLCMJ). Nevertheless, the test battery showed on average good-to-excellent intraclass correlation coefficients (0.89 < ICC < 0.97) for all tests, except for the YBT (N-Dom 0.71; LSI 0.62), SLCMJ (LSI 0.76), SLH (LSI 0.73), SH (LSI 0.41), and SJ (LSI 0.67) tests. In addition, the test battery showed on average good-to-excellent Cronbach’s α coefficients (0.80 < α < 0.98) for all tests, except for the YBT (LSI 0.70), SH (LSI 0.50), and SJ (LSI 0.74) tests.

Coefficients of variations (CV) ranged from good (YBT, 5.05%) to acceptable (SLCMJ, 26.92%) for the dominant limbs, from good (YBT, 4.74%) to not acceptable (SJ, 32.50%) for the non-dominant limbs and from good (YBT, 5.13%) to not acceptable (SH, 30.90%) for the LSI. The SJ (N-Dom, CV 32.50%) and SH (LSI, CV 30.90%) were the only tests reporting not acceptable variability. Standard errors of measurement (SEM) ranged from 0.33 (SJ) to 6.24 (SLH) for the dominant limbs from 0.52 (SJ) to 6.45 (SLH) for the non-dominant limbs and from 3.14 (YBT) to 25.04 (SH) for the LSI. Nevertheless, the most reliable value was recorded in the AT (SEM 0.25). Smallest real differences (SRD) ranged from 0.91 (SJ) to 17.24 (SLH) for the dominant limbs, from 1.44 (SJ) to 17.83 (SLH) for the non-dominant limbs and from 8.68 (YBT) to 69.20 (SH) for the LSI. All SRD values complied with the range of acceptability (SRD < 30%) except for the SH (LSI, SRD 69.20), which was considered not acceptable. Nevertheless, the best SRD value was recorded in the AT (0.69). 

As for the inter-limb asymmetries, the LSI showed a value greater than or equal to 90% (LSI ≥ 90%) for all tests in both testing sessions (T1 and T2). Average LSI ranged from 95.9 ± 6.7% (SLH) to 108.6 ± 45.3% (SH) in the first testing session (T1) and from 97.2 ± 6.9% (SLH) to 104.4 ± 12.5% (SLCMJ) in the second testing session (T2). Repeated measures ANOVA showed no significant differences (*p* > 0.05) for the LSI between the two testing sessions (T1 vs. T2). Concerning the comparisons of variances, repeated measures ANOVA showed some differences in unilateral/bilateral performance between the two testing sessions (T1 vs. T2): significant results were found for the SLH (N-Dom *p* = 0.015), SH (N-Dom *p* = 0.007), SJ (N-Dom *p* = 0.011), and LEFT (*p* = 0.009) tests. This indicates that subjects performed significantly greater with their non-dominant limb for the SLH, SH, and SJ tests while subjects performed significantly faster for the LEFT in the second testing session (T2) compared to the first testing session (T1).

## 4. Discussion

The main aim of this study was to assess test–retest reliability within a seven-day interval of an experimental test battery for the measurements of functional performance. A two-fold secondary aim was (1) to determine whether limb symmetry indices were greater than or equal to 90% (LSI ≥ 90%) in both testing sessions (T1 and T2) and (2) to establish the presence of significant performance differences between the two testing sessions (T1 vs. T2). It was hypothesized that the reliability analysis should demonstrate at least good ICC (ICC ≥ 0.75) and good Cronbach’s α (α ≥ 0.80) coefficients. The results of this study confirmed our main hypothesis, demonstrating on average a good-to-excellent test–retest reliability (0.89 < ICC < 0.97; 0.80 < α < 0.98) for the proposed functional test battery. Nevertheless, as far as the following tests are concerned, the only exceptions were observed in single dependent variables, which failed to meet the expected criteria and demonstrated a poor-to-acceptable reliability: the ICC for N-Dom in the YBT (0.71); the ICC for LSI in the YBT (0.62), SLCMJ (0.76), SLH (0.73), SH (0.41), SJ (0.67) tests, and Cronbach’s alpha for LSI in the YBT (0.70), SH (0.50), and SJ (0.74) tests.

A high reliability in the assessment of performance variables was necessary to make sound conclusions for sports injury research [41,58]. In clinical sports practice, it is essential to use reliable and objective measurements in order to conduct pre-injury screenings and monitor the rehabilitation process. The findings of the present study partially agree with those of previous research that have already investigated the reliability of different test batteries and single functional tests.

The YBT test proved to have good intra-rater reliability (ICC 0.89) and excellent interrater reliability (ICC: right leg 0.99, left leg 0.97) in male collegiate soccer players [27]. However, the latter research used a different design compared to the one adopted in the present study, namely an observation with multiple raters within a 20-min test–retest interval, with free arms during the entire YBT execution [27]. Our results in the YBT were slightly lower and showed moderate-to-good reliability (ICC: Dom 0.89, N-Dom 0.71). Nevertheless, the present study did not consider the inter-rater reliability nor such a short test–retest interval time and the YBT was executed with the arms fixed to the hips so as to prevent them from affecting the scores. Thus, the little discrepancy in the results might be associated with the differences in terms of methods used for reliability and test execution. In particular, it could be argued that a shorter test–retest time interval and the use of the arms during execution leads to higher reliability results for the YBT. Therefore, it seems that these latter aspects play a critical role for achieving greater reliability for the YBT and it is necessary to determine which method is the most valid.

A hop test battery proved to have good-to-excellent test-retest reliability (0.85 < ICC < 0.97) in ACL-injured and reconstructed athletes as a tool to discriminate between injured and uninjured limb power performances [30]. In particular, the SLCMJ (ICC 0.89) and SH (ICC 0.87) showed good reliability while the SLH (ICC 0.94) showed excellent reliability [30]. However, although the latter research used one-rater observation and performed identical test executions for the three hop tests as in the present study, a larger test–retest design was used (3–13 days interval) [30]. Our findings were slightly higher and show excellent reliability for the same hop tests: SLCMJ (ICC: Dom 0.95, N-Dom 0.93), SLH (ICC: Dom 0.93, N-Dom 0.93), and SH (ICC: Dom 0.90, N-Dom 0.92). In this specific case, study design seems to be the most relevant aspect and a shorter test–retest time interval seems to guarantee higher reliability results for the same hop tests when executed in the same way.

Another hop test battery proved to have a good-to-excellent test-retest reliability (0.84 < ICC < 0.98) and could be recommended for determining power function in healthy athletes and in the rehabilitation process [29]. More specifically, the SLCMJ (ICC: Right Leg 0.98, Left Leg 0.98) and SLH (ICC: Right Leg 0.97, Left Leg 0.97) showed excellent reliability while the SH (ICC: Right Leg 0.84, Left Leg 0.96) showed good-to-excellent reliability [29]. Our excellent reliability results for the same hop tests almost entirely agreed with those achieved by Kockum and Heijne [29], which used identical test execution for all hop tests and one-rater observation as in the present study, as much as a similar test–retest time interval of 7–10 days. Thus, it seems clear that the more similar the test–retest time intervals of two different studies are, the closer the reliability results for the same hop tests will be, provided that these are carried out with identical test execution.

Interestingly, a further functional test battery proved to have a good-to-excellent test–retest reliability (0.84 < ICC < 0.98) in uninjured and non-competitive participants as an assessment tool for decision-making on returning to sport after ACL-I [1]. Hildebrandt et al. showed good test–retest reliability (ICC: Dom 0.79, N-Dom 0.83) for the SJ using a different test execution (free arms), a shorter test–retest design (five-day interval), and the same one-rater observation compared to our study [1]. However, although a shorter test–retest time interval might guarantee higher reliability results for two different studies that assess the same hop test, the latter study showed the opposite trend: in fact, Hildebrandt et al. showed lower reliability results for the SJ compared to the current study, which demonstrated excellent test–retest reliability (ICC: Dom 0.97, N-Dom 0.96) and had a larger test–retest design (seven-day interval). Now, it could be claimed that a larger time interval (seven days) for the test–retest design is more appropriate than a shorter one (five days) to achieve higher reliability results between two different studies that use the same hop test. Seven days could be a valid solution, which is not too long nor too short for the learning effect to affect the results [38,48]. However, Hildebrandt et al. performed the SJ with free arms and not with the hands placed on the hips as in our study, and this difference in test execution might have led to the discrepancy in the results between the two studies.

All in all, the current study revealed the same good-to-excellent test–retest reliability (0.89 < ICC < 0.97) in comparison with the three test batteries mentioned hitherto [1,29,30]. However, these batteries did not contain all the tests included in the present study and carrying out a methodologically comprehensive comparison for the entire test battery remains a challenge.

The sprint tests included in this study demonstrated excellent test–retest reliability (ICC: AT, 0.95; LEFT, 0.90). Sprint tests have been shown to have excellent test–retest reliability as reported in the literature. The AT proved excellent test–retest reliability (ICC 0.96) in measuring speed and agility in collegiate men and women [35]. Nevertheless, even though Pauole et al. used the same one-rater observation as in the present study, the test–retest design used was between-trial reliability analysis (three trials) [35]. A further study showed a good-to-excellent test–retest reliability for the AT (ICC 0.82–0.96) in recreational athletes using the same test–retest design compared to our study, with a seven-day time interval and one-rater observation, but in three different testing sessions [33]. A multicenter study showed excellent reliability for the LEFT (ICC 0.96) in a student-athlete population by using identical test execution, the same test–retest time interval (seven days), and different observation with multiple raters in three testing sessions compared to our study [34]. Despite this, the divergence of the test–retest designs used in the above-mentioned studies does not seem to have affected the similarity of outcomes with the present study, by using the same test execution. Therefore, although the reliability designs were different, it seems that separate studies can achieve the same reliability results for sprint tests provided that the tests are executed in the same way.

In a nutshell, each of the tests in question proved to be highly reliable and their use in clinical sports practice combined in a test battery is highly recommended by the authors of this study. In fact, the current study proposed an experimental test battery for sports therapy, prevention, and rehabilitation purposes with a precise structure: five different unilateral tests including one for dynamic balance (YBT) and four hop tests for power functions in different plane directions (SLCMJ, SLH, SH, and SJ), which should be complemented with two sprint tests with changes of direction, one for agility (AT), and one for speed (LEFT). Furthermore, the present study attempts to fill the gap in the literature by using a specific population, a fixed test sequence, and standardized execution as well as a precise test–retest design (seven-day interval, one rater).

First, it was hypothesized that the participants involved in the present study should express a normal inter-limb symmetry range (LSI ≥ 90%) in both testing sessions (T1 and T2) as they represented an uninjured population. The results of this study confirmed our hypothesis. In fact, minimum to maximum LSI scores ranged from 95.9 ± 6.7% (SLH) to 108.6 ± 45.3% (SH) in the first testing session (T1), while from 97.2 ± 6.9% (SLH) to 104.4 ± 12.5% (SLCMJ) in the second testing session (T2). Although the LSI did not confirm our initial hypothesis for reliability, all tests showed at least moderate-to-acceptable coefficients, except for the SH test, which showed poor coefficients. For instance, the ICC for the LSI showed good reliability in the SLCMJ (0.76) test, moderate reliability in the YBT (0.62), SLH (0.73), and SJ (0.67) tests, while poor reliability in the SH (0.41) test. However, no significant differences (*p* > 0.05) were found for the LSI between the two testing sessions (T1 vs. T2), suggesting that learning effect did not have any influence on the LSI scores of two consecutive testing sessions, albeit without excellent reliability coefficients. In general, it is suggested that uninjured subjects should not exhibit inter-limb differences greater than or equal to 10% (LSI ≥ 90%) when performing functional performance tests, despite the presence of a less/more performant limb [20,21,50,59,60]. In contrast, the findings of our study advocate higher LSI ≥ 95% as the benchmark in healthy adults, indicating that the commonly accepted benchmark of LSI ≥ 90% used in clinical practice for an uninjured population may be too low. Another interesting aspect to consider is that, in the case of a dominant and operated leg, the LSI should reach a minimum of 100% to a maximum of 110% after rehabilitation [20,21,50,59,60]. However, these benchmarks (LSI 100–110%) for injured population (dominant, operated leg) were not achieved by the healthy participants included in the present study. Hence, this factor should be deeply investigated in future research.

Second, it was hypothesized that subjects should not exhibit significant differences (*p* > 0.05) in performance between the two testing sessions (T1 vs. T2). The results of this study confirmed our hypothesis. Differences were only found for single dependent variables (SLH, N-Dom, *p* = 0.015; SH, N-Dom, *p* = 0.007; SJ, N-Dom, *p* = 0.011; LEFT, *p* = 0.009), indicating that subjects performed significantly higher with their non-dominant limb (SLH, SH and SJ) and executed significantly faster (LEFT) in the second testing session (T2) compared to the first testing session (T1). The learning effect seems to have influenced the performance of the non-dominant limbs for the SLH, SH, and SJ tests in the second testing session (T2). This could be explained by the fact that optimal performance was achieved with the dominant limbs during the first testing session (T1) and was maintained stable during the second testing session (T2) for all of the tests performed. Nevertheless, performance achievements were sub-optimal during the first testing session (T1) for the non-dominant limbs and significant (*p* < 0.05) increases in performance have occurred during the second testing session (T2) for the SLH, SH, and SJ tests. Furthermore, the complexity of the execution of the LEFT seems to have played a key role in the scores obtained; plus, the learning effect seems to have led to a greater performance of the LEFT in the second testing session (T2) as subjects might have executed it faster due to their increased familiarity of the LEFT execution. However, although performance increases did occur in these tests during the second testing session (T2), the rationale remains unexplored. Therefore, the authors of the present study recommend considering the possibility of multiple tests with seven-day intervals when carrying out preventive or rehabilitative screening in clinical sports practice. This could be useful to better evaluate those tests that might be affected to a greater extent by the learning effect.

### Limitations

This study has four limitations. First, subjects were tested at a specific time of the sporting season (winter break) and they were a mixed (male and female), uninjured collegiate student-athletes’ population with a large age range (18–30 years). Additionally, subjects came from both team and individual sports and none competed at a professional level but were all involved at a competitive and regional level. Second, the influence of growth and maturation status, practiced sport, and gender on the test results was not considered. Third, the between-session reliability with one rater was the only type of analysis considered, while intra-rater and interrater reliability analyses were not. Fourth, the proposed baseline values refer to a small number of participants and our results can only be applied to a population of healthy adults.

## 5. Conclusions

The experimental test-battery proposed in this study appears to be highly reliable (ICC ≥ 0.75; Cronbach’s α ≥ 0.80) for the measurements of functional performance in healthy adults. Thus, the implementation of its standardized test protocol in sports clinical practice is strongly recommended by the authors for prevention and rehabilitation purposes. Furthermore, if performed on an uninjured population, a normal inter-limb symmetry range (LSI ≥ 90%) can be expected for all unilateral tests. The findings also suggest that the benchmark for clinical practice can be set at LSI ≥ 95%. However, subjects performed significantly greater in three hop tests with the non-dominant limb (SLH, *p* 0.015; SH, *p* 0.007; SJ, *p* 0.011) and performed significantly faster in one sprint test (LEFT, *p* 0.009) in the second testing session compared to the first testing session. Therefore, an improvement in performance due to the learning effect can be expected in these specific tests and future studies should provide a more in-depth analysis of these aspects.

## Figures and Tables

**Table 1:** Anthropometric data.

	Male (Mean ± SD)	Female (Mean ± SD)	Total (Mean ± SD)
**Number**	14	8	22
**Age (years)**	23.6 ± 2.9	22.8 ± 5.4	23.3 ± 3.9
**Mass (kg)**	78.4 ± 6.1	65.9 ± 7.3	73.8 ± 8.6
**Height (m)**	1.80 ± 0.1	1.71 ± 0.1	1.77 ± 0.1
**BMI (kg/m^2^)**	24.1 ± 1.5	22.6 ± 2.3	23.6 ± 1.9
**Limb length (cm)**	N-Dom 100.9 ± 4.3	N-Dom 97.3 ± 4.9	N-Dom 99.6 ± 4.8
	Dom 100.9 ± 4.3	Dom 97.3 ± 4.7	Dom 99.6 ± 4.7

SD = standard deviation; N-Dom = non-dominant leg; Dom = dominant leg; kg = kilograms; m = meters; cm = centimeters; BMI = body mass index.

**Table 2:** Results of the test battery.

Test	Session 1 (Mean ± SD)	Session 2 (Mean ± SD)	ANOVA (*p* < 0.05)	Cronbach’s Alpha (α)	ICC (95% CI)	CV	SEM	SRD
**YBT (cs)**								
Dom	86.8 ± 4.7	87.4 ± 4.1	0.273	0.92	0.89 (0.86–0.92)	5.05	1.46	4.03
N-Dom	85.8 ± 4.6	87.2 ± 3.5	0.081	0.80	0.71 (0.62–0.80)	4.74	2.21	6.11
LSI (%)	99.0 ± 5.6	99.9 ± 4.6	0.393	0.70	0.62 (0.53–0.70)	5.13	3.14	8.68
**SLCMJ (cm)**								
Dom	15.5 ± 4.3	15.8 ± 4.2	0.380	0.96	0.95 (0.93–0.96)	26.92	0.94	2.60
N-Dom	15.5 ± 4.5	16.2 ± 4.1	0.078	0.95	0.93 (0.90–0.95)	27.04	1.14	3.15
LSI (%)	101.3 ± 14.1	104.4 ± 12.5	0.164	0.83	0.76 (0.70–0.82)	12.94	6.52	18.02
**SLH (cm)**								
Dom	139.6 ± 24.2	143.5 ± 23.3	0.071	0.96	0.93 (0.91–0.95)	16.68	6.24	17.24
N-Dom	134.0 ± 25.0	139.4 ± 23.8	0.015 *	0.96	0.93 (0.90–0.95)	17.78	6.45	17.83
LSI (%)	95.9 ± 6.7	97.2 ± 6.9	0.289	0.80	0.73 (0.66–0.80)	7.04	3.53	9.76
**SH (no.)**								
Dom	54.7 ± 15.2	57.3 ± 13.0	0.116	0.94	0.90 (0.85–0.92)	25.00	4.46	12.33
N-Dom	54.9 ± 11.0	58.0 ± 11.6	0.007 *	0.95	0.92 (0.90–0.94)	19.87	3.19	8.82
LSI (%)	108.6 ± 45.3	102.3 ± 9.8	0.447	0.50	0.41 (0.31–0.50)	30.90	25.04	69.20
**SJ (s)**								
Dom	7.9 ± 1.7	7.7 ± 2.1	0.180	0.98	0.97 (0.96–0.98)	24.36	0.33	0.91
N-Dom	8.3 ± 2.8	7.8 ± 2.4	0.011 *	0.98	0.96 (0.95–0.97)	32.50	0.52	1.44
LSI (%)	103.4 ± 13.8	100.7 ± 8.3	0.246	0.74	0.67 (0.60–0.73)	11.17	6.54	18.07
**AT (s)**	11.6 ± 1.2	11.7 ± 1.0	0.528	0.96	0.95 (0.93–0.96)	9.40	0.25	0.69
**LEFT (s)**	110.8 ± 11.4	107.7 ± 10.3	0.009 *	0.94	0.90 (0.86–0.92)	9.97	3.45	9.53

Dom = dominant leg; N-Dom = non-dominant leg; LSI = limb symmetry index; SD = standard deviation; cs = composite score; cm = centimeter; no. = number; s = seconds; ICC = intraclass correlation coefficient; CV = coefficient of variation; SEM = standard error of measurement; SRD = smallest real difference; YBT = Y-Balance Test; SLCMJ = Single-Leg Countermovement Jump Test; SLH = Single-Leg Hop for Distance test; SH = Side-Hop Test; SJ = Speedy-Jump Test; AT = Agility *T-*Test; LEFT = Lower Extremity Functional Test. * = significant difference in performance between testing sessions; ICC values in parenthesis are 95% confidence interval.

## Data Availability

The data presented in this study are available on request from the corresponding author. The data are not publicly available due to privacy restrictions.
